# An intravaginal ring that releases three antiviral agents and a contraceptive blocks SHIV-RT infection, reduces HSV-2 shedding, and suppresses hormonal cycling in rhesus macaques

**DOI:** 10.1007/s13346-017-0389-0

**Published:** 2017-06-09

**Authors:** Nina Derby, Meropi Aravantinou, Jessica Kenney, Shweta R. Ugaonkar, Asa Wesenberg, Jolanta Wilk, Larisa Kizima, Aixa Rodriguez, Shimin Zhang, Olga Mizenina, Keith Levendosky, Michael L. Cooney, Samantha Seidor, Agegnehu Gettie, Brooke Grasperge, James Blanchard, Michael Piatak, Jeffrey D. Lifson, José Fernández-Romero, Thomas M. Zydowsky, Melissa Robbiani

**Affiliations:** 1 Population Council, 1230 York Avenue, New York, NY 10065 USA; 2 Aaron Diamond AIDS Research Center, 455 First Avenue, 7th Floor, New York, NY 10016 USA; 3 Tulane Primate Research Center, 18703 Three Rivers Road, Covington, LA 70433-8915 USA; 4 AIDS and Cancer Virus Program, Leidos Biomedical Research, Inc., Frederick National Laboratory for Cancer Research, Frederick, MD 21702-1201 USA; 5 Science Department, Borough of Manhattan Community College, The City University of New York, 199 Chambers Street, New York, NY 10007 USA

**Keywords:** Multipurpose prevention technology, Intravaginal ring, HIV, HSV-2, HPV, Contraception

## Abstract

Women globally need access to multipurpose prevention technologies (MPTs) that prevent human immunodeficiency virus (HIV), sexually transmitted infections that increase HIV acquisition/transmission risk, and unintended pregnancy. Seeking an MPT with activity against HIV, herpes simplex virus-2 (HSV-2), and human papillomavirus (HPV), we developed a prototype intravaginal ring (IVR), the MZCL IVR, which released the antiviral agents MIV-150, zinc acetate, and carrageenan (MZC for short) and the contraceptive levonorgestrel (LNG). Previously, we showed that an MZC gel has potent activity against immunodeficiency viruses, HSV-2, and HPV and that the MZCL (MZC with LNG) IVR releases all four components in macaques in vivo at levels associated with efficacy. Vaginal fluid from treated macaques has in vitro activity against HIV, HSV-2, and HPV. Herein, we assessed the ability of the MZCL IVR to protect macaques against repeated co-challenge with HSV-2 and SHIV-RT (simian immunodeficiency virus [SIV] containing the reverse transcriptase gene from HIV) and prevent hormonal cycling. We evaluated in vivo drug release in co-challenged macaques by measuring drug levels in blood and vaginal fluid and residual drug levels in used IVRs. The MZCL IVR significantly prevented SHIV-RT infection, reduced HSV-2 vaginal shedding, and prevented cycling. No non-nucleoside HIV reverse transcriptase inhibitor (NNRTI)-resistant SHIV was detected in macaques that became infected after continuous exposure to MZC from the IVR. Macaques wearing the MZCL IVR also had carrageenan levels in vaginal fluid expected to protect from HPV (extrapolated from mice) and LNG levels in blood associated with contraceptive efficacy. The MZCL IVR is a promising MPT candidate that warrants further development.

## Introduction

The non-curable sexually transmitted infections (STIs) caused by human immunodeficiency virus (HIV), herpes simplex virus type 2 (HSV-2), and human papillomavirus (HPV), as well as unintended pregnancy, are highly overlapping global risks for women’s health that converge most significantly in sub-Saharan Africa [1]. A safe, acceptable, affordable, and accessible self-initiated multipurpose prevention technology (MPT) that protects women against these four indications could significantly improve the health of women globally.

A large number of topically applied vaginal formulations have been developed to meet end-user preferences for HIV prevention [2, 3]. These formulations can be broadly categorized as short-acting/on-demand (e.g., douches, films, gels, inserts, nanofibers) and long-acting/sustained release (e.g., intravaginal rings [IVRs]). Several gels and IVRs that release one or more anti-HIV drugs have demonstrated their potential utility by blocking immunodeficiency virus infection in preclinical models [2, 3] and have advanced beyond Phase 1 clinical testing.

However, the microbicide gels tested in Phase 3 clinical trials failed to live up to expectations, even though early clinical phase data looked encouraging (especially for 1% tenofovir gel tested in the CAPRISA-004 2B trial) [4, 5]. Subset analysis of these Phase 3 gel trials concluded that poor adherence to gel confounded the results. However, women who consistently used the gels were at a lower risk of acquiring HIV infection [6]. The two recently completed Phase 3 trials of the dapivirine IVR showed that the IVRs significantly reduced the risk of HIV infections overall [7]. Similar to the gel trials, poor adherence to IVR use decreased the overall efficacy of the IVR, and in some subgroups (notably the youngest women), the IVR did not protect [7]. These data support the notion that women who consistently use topical microbicides will likely be protected from HIV.

Therefore, the next generation(s) of topical microbicides must be designed to encourage greater adherence to study product. One avenue to achieving this is to broaden the utility and appeal of the product by expanding its indications to include other STIs and unintended pregnancy. IVRs are uniquely able to address multiple sexual and reproductive health needs as they are the leading sustained release platform to deliver multiple drugs vaginally, have high acceptability, and are already on the market for contraception as well as hormone replacement therapy and preparation for in vitro fertilization [8, 9]. MPT IVRs that prevent pregnancy and STIs may improve microbicide usage, increasing coverage and effectiveness and significantly reducing global health burdens. An MPT IVR that covers multiple indications, including HIV, other STIs, and contraception, may have added value and also reduce the overall cost of prevention by providing the multiple indications from one product. Although IVRs are more expensive than some other forms of pre-exposure prophylaxis (PrEP) per unit, the overall cost savings and impact on global public health through increased adherence and sustained delivery will likely reduce costs long-term and make IVRs competitive with other dosage forms.

The Population Council has developed the MZC combination microbicide as an MPT to simultaneously prevent HIV-1, HSV-2, and HPV infections. It is composed of three antiviral drugs: MIV-150, a non-nucleoside HIV reverse transcriptase inhibitor (NNRTI); zinc acetate (ZA), a small molecule metal salt; and carrageenan (CG), a high molecular weight naturally occurring sulfated polysaccharide. MZC blocks HIV, HSV-2, and HPV through multiple mechanisms of action [10]. MIV-150 targets different clades and drug-resistant isolates of HIV [10], and ZA targets HIV and HSV-2 [10–14], potentially recognizing an RT site in HIV distinct from that recognized by MIV-150 [11]. CG potentiates ZA’s anti-HSV-2 activity [12, 14], potently blocks HPV in mice and macaques [10, 15–17], and reduces the prevalence of HPV in women [18]. Formulated as a semisolid aqueous gel, the MZC combination significantly reduces (i) macaque SHIV-RT infection after SHIV-RT or SHIV-RT/HSV-2 challenge [10, 19–21], (ii) HSV-2 shedding in macaques after repeated SHIV-RT/HSV-2 challenge [13], and (iii) infection of mice with HSV-2 and HPV [10]. Levonorgestrel (LNG) is used in many licensed contraceptives, is on the WHO list of essential medicines [22], and is currently the top contender for inclusion in vaginally applied microbicide/contraceptive products [9]. Thus, MZCL could prevent the three viral infections and unintended pregnancy. Both the MZC and MZCL IVRs could have better adherence due to their multiple indications.

We tested the MZC and MZCL IVRs in a repeated SHIV-RT/HSV-2 co-challenge rhesus macaque model that we recently developed [20]. Non-depot medroxyprogesterone acetate (DMPA)-treated macaques received twice-weekly vaginal challenges containing 200 TCID_50_ SHIV-RT and 10^7^ pfu HSV-2 for 10 weeks (wks). Both viruses infect the animals at a frequency similar to that observed in DMPA-treated macaques co-challenged once with 1000 TCID_50_ SHIV-RT and 2 × 10^8^ pfu HSV-2 [20]. This dosing regimen results in a similar pattern of SHIV viremia but more frequent HSV-2 shedding in the vaginal fluid, which provides power to detect effects of the microbicide on HSV-2 shedding in infected macaques as well as on outright infection.

Herein, we performed basic pharmacokinetics (PK) testing on a larger sample size than that reported before to substantiate the efficacy results and to inform future IVR optimization, evaluated antiviral efficacy against the acquisition of SHIV-RT and HSV-2 infections, assessed vaginal HSV-2 shedding, examined correlates of the contraceptive activity of LNG, and looked for the emergence of drug resistance in macaques that became infected during the study. Sustained use of an MZC or MZCL IVR by an HIV-infected woman who is either unaware of her HIV status or interested in the additional protection provided by the IVR could potentially lead to the emergence of common NNRTI-resistance mutations. We provide the first evidence that an IVR delivering the unique broad-spectrum MZC combination microbicide and the contraceptive LNG significantly protects macaques against repeated vaginal challenge with SHIV-RT without leading to NNRTI resistance in breakthrough infections, significantly reduces HSV-2 shedding, produces vaginal levels of CG associated with protection from HPV, and suppresses macaque menstrual cycles. An MPT IVR that significantly reduces the sexual transmission of HIV, the transmission and viral burden of STI co-factors for HIV acquisition like HSV-2 and HPV, and the risk of unintended pregnancy could improve the sexual and reproductive health of millions of women worldwide.

## Materials and methods

### IVRs

**Fig. 1:**
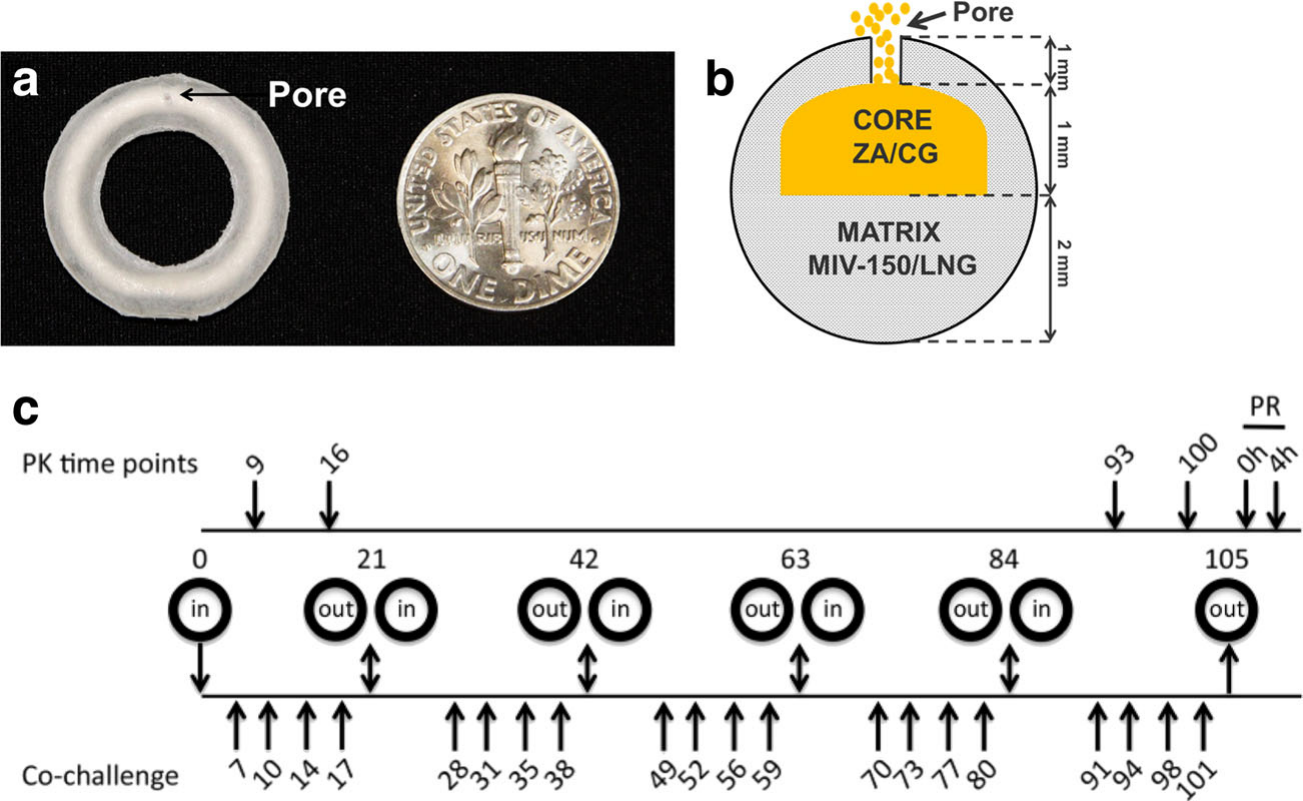
Design of the macaque MZCL IVR study. (**a**, **b**) Graphical representation of the MZCL IVR. The thermoplastic matrix containing MIV-150 and LNG encompasses the compressed core of CG and ZA. A pore (500 or 800 μm) drilled through the matrix to the core exposes the core to incoming vaginal fluid, allowing hydration of the core and release of CG and ZA. (**c**) A repeated SHIV-RT/HSV-2 co-challenge model was used for testing the MZCL IVR. IVRs were inserted into rhesus macaques not treated with DMPA for 21 d before they were exchanged for new IVRs for a total of 5 IVR cycles over 105 d. Co-challenge with 200 TCID_50_ SHIV-RT and 10^7^ pfu HSV-2 occurred on d7, 10, 14, and, 17 of each IVR cycle. PK time points were on d9 and d16 of the first (IVR-1) and fifth (IVR-5; this corresponds to d93 and d100 of the entire study for IVR-5) IVR cycles as well as post-removal (PR) of IVR-5 (0 h PR). Blood was collected at additional time points post-insertion of IVR-1 and IVR-5 and also 4 h PR of IVR-5

A complete description of the design, manufacturing, and characterization of the core-matrix MZC and MZCL IVRs and the matrix placebo and LNG IVRs has been published [23]. Briefly, MZC(L) IVRs (20 mm × 4 mm) consisted of a solid compressed core of ZC encased by an ethylene vinyl acetate (thermoplastic) ring body/matrix that contained MIV-150 and LNG (in MZCL IVRs). A pore was drilled into the IVR matrix to allow ZC gel (formed by the influx of vaginal fluid via the pore to hydrate the solid ZC core) to exit. MIV-150 and LNG diffused through the thermoplastic. All IVRs were subjected to quality control testing prior to use in the animals. The tests included microscopic examination of the pore to determine pore size and also to ensure that the pores were not obstructed in any way due to the fabrication process. The quality control procedures are described in Ugaonkar et al. [23]. A graphical representation of the IVR is shown in Fig. 1a, b. MZC and MZCL IVRs with two pore sizes—500 and 800 μm—were tested.

### Viruses

SHIV-RT for macaque in vivo challenges was grown in phytohaemagglutinin (PHA)-activated rhesus macaque PBMCs and titered on 174xCEM (CX1) cells as described [21]. This stock, previously used in another study [19], had a wild type RT gene sequence in 32 of 32 clones sequenced (not shown). In drug-resistance studies, PBMC virus from macaque EJ42 was expanded by 8 days (d) of co-culture with CX1 cells (5 × 10^5^ PBMC:5 × 10^5^ CX1) in 24-well plates. The culture supernatant was clarified by centrifugation (10,000 ×*g* for 15 minutes [min]) and frozen at −80 °C before titering using the TZM-bl assay [24]. For comparison, SHIV-RT was expanded alongside EJ42 PBMC virus by spinoculation of CX1 cells with 400 TCID_50_/10^6^ cells in 96-well flat-bottom plates (1700 ×*g*, 100 min, 23 °C) followed by 8 d of reculture in 24-well plates. Virus was collected in the culture supernatant and titered on TZM-bl cells as described above. HIV-1_NL43_ I178V was constructed by site-directed mutagenesis (Genewiz, South Plainfield, NJ) on the HIV-1_NL43_ plasmid, and the virus was generated by transfection of 293T cells and titered on TZM-bl cells as above. The virus stock was titered using 3 × 3 activated human PBMCs [25]. TCID_50_ was calculated using the Reed and Muench formula. Aliquots of virus stock were stored at −80 °C. HSV-2 strain G (ATCC) was grown and titered in Vero cells as previously described [26].

### Macaque studies

#### Ethics statement

The TNPRC Institutional Animal Care and Use Committee (IACUC) board granted approval for the macaque studies. Adult female rhesus macaques (*Macaca mulatta*) were housed and cared for at Tulane National Primate Research Center (TNPRC, Covington, LA) in accordance with the policies of the TNPRC Animal Care and Use Committee (OLAW Assurance A4499-01), which is accredited by the Association for Assessment and Accreditation of Laboratory Animal Care (AAALAC 000594). All procedures complied with the Animal Welfare Act [27], the *Guide for the Care and Use of Laboratory Animals* [28], and TNPRC standards for minimizing animal distress. Macaques were socially housed indoors in climate-controlled conditions with a 12/12-light/dark cycle and monitored for their welfare continuously throughout the study. Macaques were anesthetized with ketamine-HCl (10 mg/kg) or tiletamine/zolazepam (6 mg/kg) before all procedures, and pre-emptive and post-procedural buprenorphine (0.01 mg/kg) were given for procedures that would likely cause more than momentary pain or distress in humans undergoing the same procedures. One macaque (CT31) stopped eating and lost weight 6 wks after the last co-challenge and was euthanized using methods consistent with recommendations of the American Veterinary Medical Association Panel on Euthanasia; it was anesthetized with tiletamine/zolazepam (8 mg/kg intramuscularly [im]) and given buprenorphine (0.01 mg/kg im) followed by an overdose of pentobarbital sodium. Death was confirmed by auscultation of the heart and pupillary dilation. All other macaques remained healthy and were released at the end of the study to be enrolled in other microbicide studies.

#### Study design

As described in Table 1, there were four major groups of macaques for comparison in this study: those that received placebo IVRs (*n* = 4), LNG IVRs (*n* = 4), MZC IVRs (*n* = 12), and MZCL IVRs (*n* = 12). In the test groups (MZC and MZCL), we compared IVRs with a 500 μm pore (*n* = 6 MZC and 6 MZCL) and an 800 μm pore (*n* = 6 MZC and 6 MZCL). As we previously reported [23], IVRs with the 800 μm pore had the pore plugged with ZA/CG mix to provide a continuum between the IVR core and the vaginal fluid. In the placebo and LNG control groups, 500 and 800 μm pore were included but without plugging since these IVRs were of matrix design and did not contain a core; thus, the pore was superficial. The study was conducted during the October to April breeding season for rhesus macaques when the animals are cycling. Macaques were confirmed negative for SIV, simian type D retroviruses, simian T cell leukemia virus-1, and herpes B and were assigned to unblinded treatment groups that were not randomized since there was no screening data on their human leukocyte antigen types or intrinsic antiviral gene alleles. Staff used visual inspection with a speculum at every challenge and time point to confirm that each animal retained the IVR. MZC and MZCL IVRs were compared to placebo and LNG IVRs by using a modified version of our recently published, repeated (weekly for 20 wks) SHIV-RT/HSV-2 co-challenge model in non-DMPA-treated rhesus macaques [20]. Based on data from the published PK study on these IVRs [23], we modified the co-challenge regimen to focus on a challenge window when vaginal fluid drug levels were >25 μg/ml CG (effective against HPV and HSV-2 [10, 17, 29]) and >50 ng/ml MIV-150 (effective against SHIV without being at peak levels [19, 21]). IVRs (IVR-1) were vaginally inserted into cycling macaques with the pore facing the cervix, and on d7 post-insertion (PI), the macaques were co-challenged with 200 TCID_5__0_ SHIV-RT and 10^7^ pfu HSV-2. Additional co-challenges were administered on d10, 14, and 17. On d21, the used IVRs were replaced with fresh IVRs (IVR-2), and co-challenge was initiated again on d7, 10, 14, and 17 post-insertion. This regimen was repeated for a total of 5 IVR cycles and 20 co-challenges over 105 d. Vaginal fluid was collected for PK only on d9 and d16 of the first (IVR-1) and fifth (IVR-5) cycles as well as immediately post-removal (PR) of IVR-5 (0 h PR) to minimize disturbances to the vaginal environment; this corresponds to d9, d16, d93, and d100 of the entire study. Blood was collected at additional time points after insertion of IVR-1 and IVR-5 and also 4 h after removal of IVR-5. Cervical and vaginal pinch biopsies were collected 9 wks after the last co-challenge. The study design, including these PK time points, is illustrated in Fig. 1c.

**Table 1:** Macaques treated with MZC and MZCL IVRs

Animal ID	IVR	Pore	SHIV-RT		HSV-2 infection^b^
		Size (μm), ±plug	Infection	Ab^a^	
CT31	LNG	500, no plug	Ch 5^c^	Ch 9	+
GD55	LNG	500, no plug	Ch 6	Ch 9	+
HA51	LNG	800, no plug	Ch 3	Ch 6	+
HD87	LNG	800, no plug	Ch 5	Ch 8	+
HH91	MZCL	500, no plug	−	−	+
GI11	MZCL	500, no plug	−	−	−
FC81	MZCL	500, no plug	−	−	+
FI10	MZCL	500, no plug	−	−	−
GK45	MZCL	500, no plug	Wk 3	Wk 8^d^	+
GA17	MZCL	500, no plug	−	−	+
GC05	MZCL	800, plug	Wk 4	Wk 8^d^	−
ID23	MZCL	800, plug	−	−	−
IC87	MZCL	800, plug	Wk 1	Wk 3^d^	+
HI81	MZCL	800, plug	−	−	+
HN43	MZCL	800, plug	Ch 13	Ch 14	+
EL86	MZCL	800, plug	−	−	−
FH29	Placebo	500, no plug	−	−	+
HH78	Placebo	500, no plug	−	−	+
GB85	Placebo	800, no plug	Ch 16	Ch 17	+
EA90	Placebo	800, no plug	Ch 5	−	+
GP71	MZC	500, no plug	−	−	+
FV47	MZC	500, no plug	−	−	+
HN94	MZC	500, no plug	−	−	+
HT57	MZC	500, no plug	−	−	+
DT20	MZC	500, no plug	−	−	+
EJ42	MZC	500, no plug	Ch 5	Ch 7	+
HB13	MZC	800, plug	−	−	+
GI81	MZC	800, plug	−	−	+
IT26	MZC	800, plug	Ch 19	Wk 3^d^	+
IT36	MZC	800, plug	−	−	+
HV50	MZC	800, plug	−	−	+
GA74	MZC	800, plug	−	−	−

^a^Antibody

^b^Infection status determined from HSV-2 DNA in vaginal fluid and vaginal and cervical tissues

^c^CT31 was euthanized 6 weeks post-last co-challenge because it met TNPRC IACUC endpoint criteria

^d^Preceding week’s sample not available

#### Sampling

Blood (<10 ml/kg/month), vaginal fluid, and cervical and vaginal pinch biopsies were collected as previously described [30] and shipped overnight from TNPRC to the Population Council’s NYC laboratories. Vaginal fluid was collected using a swab, which was subsequently immersed in 1 ml saline. This sample contained vaginal fluid and cells since it was not centrifuged to prevent CG from settling. As we previously described [30], plasma and peripheral blood mononuclear cells (PBMCs) isolated from blood and vaginal fluid samples were mixed, aliquotted, and frozen at −80 °C.

### Pharmacokinetics

MIV-150 in vaginal fluid was measured by radioimmunoassay (RIA, LLOQ = 1 ng/ml) [10] and in plasma by using liquid chromatography with tandem mass spectrometry (LCMS/MS, LLOQ = 20 pg/ml) [21]. CG in vaginal fluid was measured by ELISA (LLOQ = 40 ng/ml) [10]. LNG in serum was measured by RIA (Immunometrics Ltd., London, UK) at the Oregon National Primate Research Center (ONPRC), Endocrine Technology and Support Core Laboratory (ETSC, Beaverton, OR) [23]. The range of detection of the LNG RIA was 23–375 fmol/sample with a sensitivity of 36–47 pg/ml. Samples were analyzed in duplicate. ZA in vaginal fluid was not quantified due to the lack of a validated assay. However, residual ZA remaining in IVRs after 21d in vivo was quantified as described in the next section.

### Drug levels in used IVRs

Amounts of the four drugs remaining in IVRs 1 and 5 after 21d in vivo were quantified as previously described [23]. Briefly, IVRs were cut in half and core material was eluted with 10 ml acetate buffer, which was analyzed for CG and ZA content. Residual MIV-150 and LNG in the IVR matrix were extracted with dichloromethane, which was analyzed via high-pressure liquid chromatography (HPLC).

### Endogenous hormones

Serum levels of estradiol and progesterone were determined at the ONPRC ETSC using a chemiluminescence-based automatic clinical platform (model Cobas e411, Roche Diagnostics, Indianapolis, IN) validated for estradiol (sensitivity, range 5–4300 pg/ml) and progesterone (sensitivity range 0.03–60 ng/ml) in monkey serum [31].

### Virus detection

Quantitative reverse transcription-polymerase chain reaction (qRT-PCR) was used to measure SIV *gag* RNA in plasma (LLOQ = 15–30 copies/ml) [30]. ELISA was used to measure antibodies directed to SIV in plasma [13]. Viral DNA for HSV-2 detection was extracted from the cell/fluid vaginal fluid mixture using the QIAamp DNA blood mini kit (Qiagen, Valencia, CA) or from cervical or vaginal biopsies using the DNeasy blood and tissue kit (Qiagen) according to the manufacturer’s instructions and as previously described [13]. Six independent nested PCRs on a segment of the *gD* gene were performed for each macaque at each time point and run on agarose gels. The number of positive reactions of 6 total was scored. The identity of the HSV-2 gD amplicon was confirmed by sequencing. Time points at which HSV-2 DNA was not detectable in any nested PCR reaction were subjected to GAPDH PCR to confirm DNA quality.

### RT sequencing

The RT gene from the blood of macaques that became infected while wearing a MIV-150-containing IVR was sequenced as described [32]. Plasma virus was sequenced during acute infection or a time point after the conclusion of all challenges when the tested volume contained ≥10,000 copies of virus, unless no such sample was available. From macaque EJ42, we also sequenced PBMC virus from chronic infection. All viruses tested in vitro against NNRTIs were sequenced prior to testing.

### Antiviral properties of active pharmaceutical ingredients in the presence of biological fluids

The antiviral activity of diluted macaque vaginal fluids (see Sampling section) was tested in the TZM-bl assay as previously described [30]. Virus inoculum for the assay was prepared in cell culture medium plus or minus 25% whole pooled human semen (Lee Biosolutions, St. Louis, MO). The previously described antiviral assay was used to evaluate the sensitivity of viruses (expanded EJ42 PBMC virus, HIV-1_NL43_ I178V, HIV-1_NL43_ wild type, and SHIV-RT) to NNRTIs (MIV-150, rilpivirine, etravirine, and efavirenz) [24].

### Statistical analysis

Data were analyzed using GraphPad Prism v5.0c (GraphPad Software, San Diego, CA) and SAS (Cary, NC). Macaque SHIV infection frequency was analyzed using the Peto-Peto-Prentice test to determine significance of treatment with pairwise comparisons made using Sidak’s adjustment. Macaque HSV-2 infection and shedding were analyzed with a logistic mixed model using the *F* test for overall effect of treatment and pairwise comparisons made by Scheffé-adjusted *t* tests. Comparisons between groups in PK studies were made using a log-normal generalized linear mixed model. Overall effects were tested with the *F* test and pairwise comparisons made with Scheffé-adjusted *t* tests. Drugs remaining in used IVRs were analyzed with a beta generalized linear mixed model. Overall effects were tested with *F* tests and pairwise comparisons (for MIV-150) made with *t* tests adjusted by simulation. In vitro anti-HIV activity was analyzed by Mann–Whitney *U* test. Associations between variables were tested with Spearman correlation analysis. Significance was defined by *p* < 0.05. For each comparison where possible, the ratio, odds ratio (OR), or Spearman *r* value is reported alongside its 95% confidence interval (CI). Due to small sample sizes, power in this study is low although unquantifiable due to the use of generalized linear mixed models incorporating repeated measures and random effects.

## Results

### In vitro release of MZCL in vivo from a core-matrix IVR

**Fig. 2:**
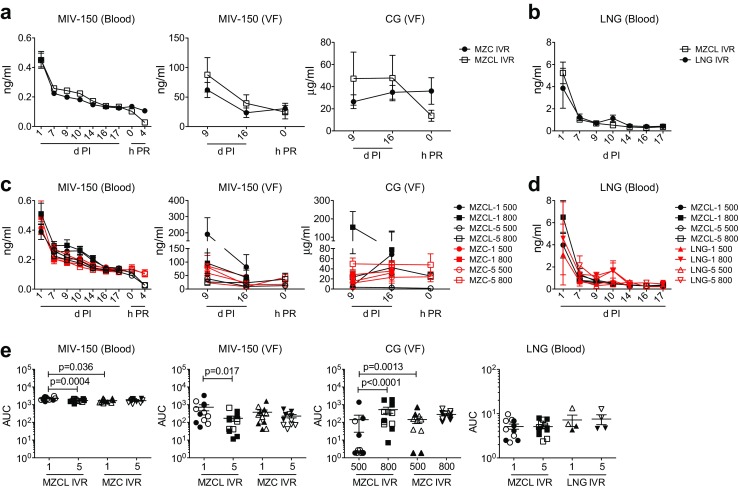
Drug release from novel core-matrix MZC and MZCL IVRs in vivo. This figure details the PK profile of the MZCL vs. MZC IVRs overall (**a**, **b**) and per particular IVR design (**c**, **d**, **e**). (**a**) At the indicated times post-insertion (PI) and post-removal (PR) in macaques carrying MZCL or MZC IVRs, MIV-150 was measured in blood and vaginal fluid (VF), and CG was measured in vaginal fluid. (**b**) LNG was similarly measured in the blood of macaques carrying MZCL or LNG IVRs. To show the overall difference between MZCL and MZC IVRs, in (**a**) and (**b**), data were pooled from macaques carrying IVRs of different pore sizes and from IVR cycles 1 and 5. Thus, mean ± SEM is shown for 24 MZCL IVRs (12 from IVR-1 and 12 from IVR-5), 24 MZC IVRs (12 from IVR-1 and 12 from IVR-5), and 8 LNG IVRs (4 from IVR-1 and 4 from IVR-5). The breakdown by pore size and IVR cycle (*n* = 6 each) is shown for (**c**) MIV-150 and CG and (**d**) LNG. (**e**) The total amounts of MIV-150, CG, and LNG detected in vivo were determined by analyzing area under the curve (AUC) of the data in (**c**) and (**d**). For MIV-150 and LNG, *closed symbols* represent 500 μm pore IVRs and *open symbols* represent 800 μm pore IVRs. For CG, *closed symbols* represent IVR-1 and *open symbols* represent IVR-5

**Fig. 3:**
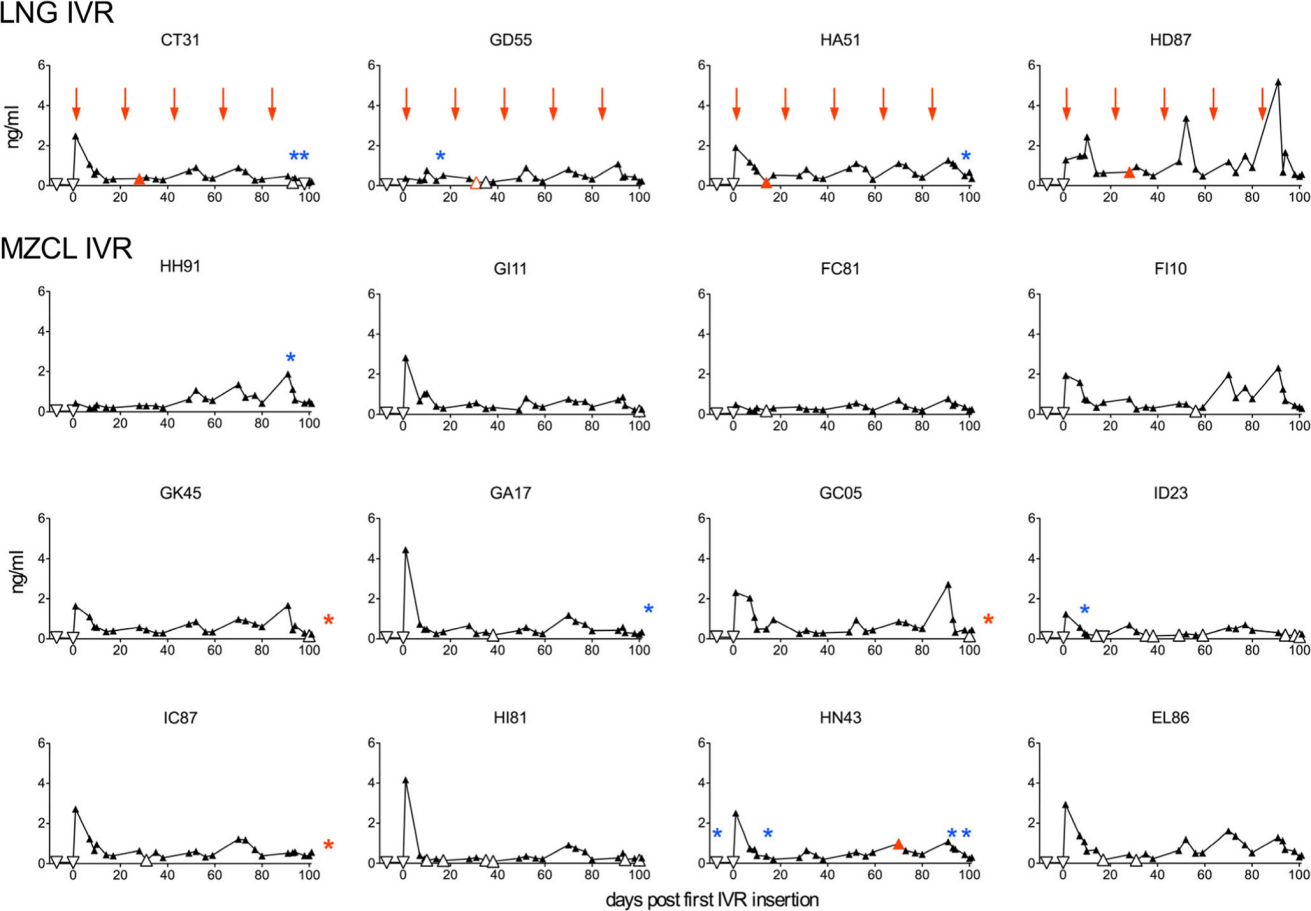
Levels of LNG released from MZCL IVRs were not impacted by MZC. Levels of LNG in blood resulting from in vivo release from LNG and MZCL IVRs are shown. *Arrows* indicate times IVRs were inserted/exchanged. *Open triangles* represent LNG levels between 0.10 and 0.19 ng/ml. *Open inverted triangles* represent LNG levels between 0.09 and 0.10 ng/ml. The first SIV RNA positive time point for each animal is shown *in red with red asterisks* indicating that plasma virus RNA was only detected after challenges were concluded. *Blue asterisks* indicate times of noticeable vaginal bleeding

**Fig. 4:**
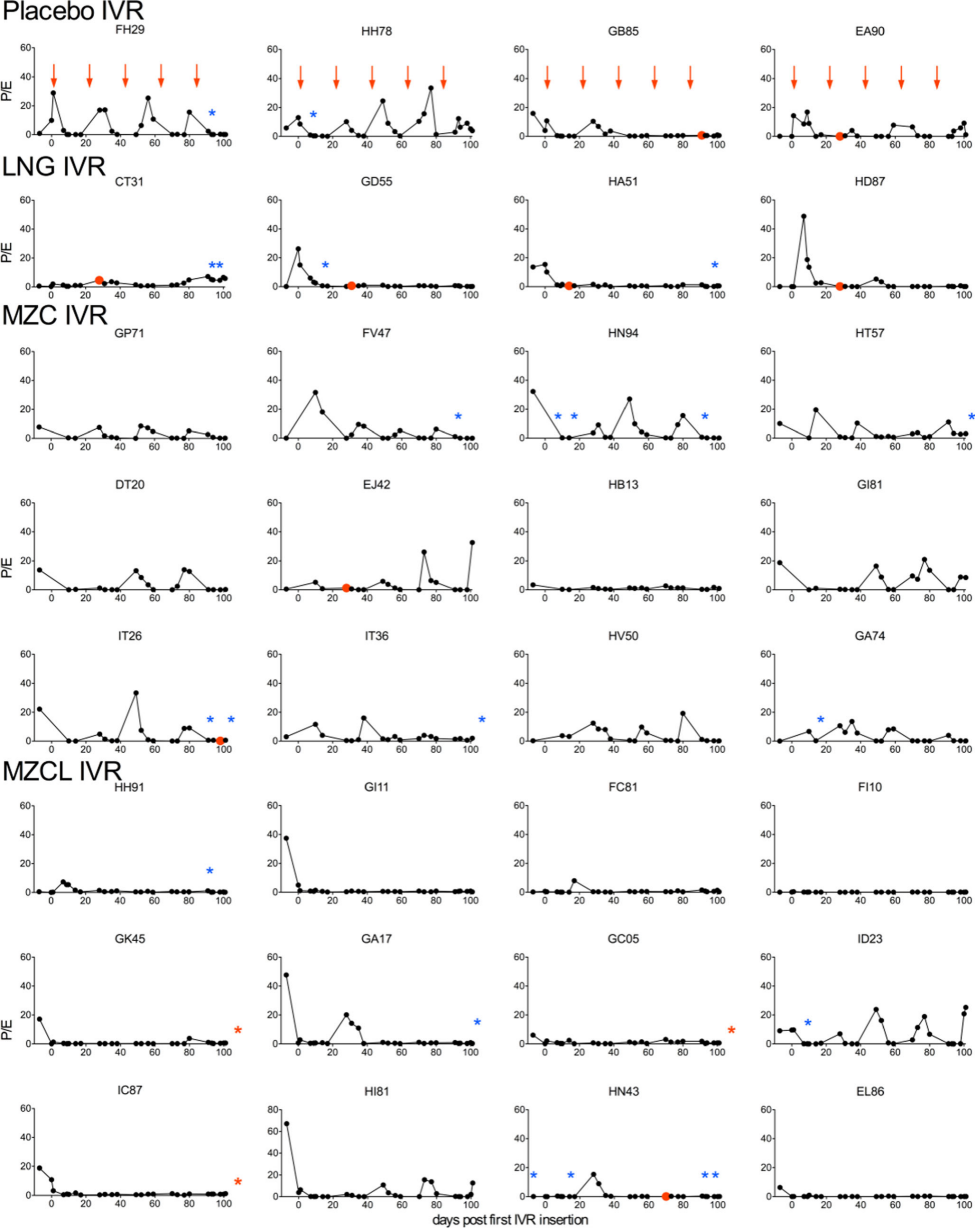
LNG released from IVRs suppressed normal cycling in macaques. Estradiol-17 (E, pg/dl) and progesterone (P, ng/ml) were measured in blood over time. *Arrows* indicate times IVRs were inserted/exchanged. The first SIV RNA positive time point for each animal is shown in *red with red asterisks* indicating that plasma virus RNA was only detected after challenges were concluded. *Blue asterisks* denote noticeable vaginal bleeding

Fig. 1a, b show the design of the IVRs used in this study, and Fig. 1c shows the macaque study design. To monitor IVR function, obtain additional PK data from virus-challenged macaques, and compare data from this study with our initial results [23], we measured drug levels in blood and vaginal fluid at select time points during the first (IVR-1) and fifth (IVR-5) IVR cycles as well as residual drug content in these IVRs after removal. MIV-150 was detected both in blood and vaginal fluid, and in both matrices the concentrations dropped over time (Fig. 2a). LNG blood levels met or exceeded the target, 0.19 ng/ml [23, 33–35], in all macaques (Fig. 2b), occasionally dropping to ≥0.09 ng/ml after d10. Low LNG blood levels were not associated with a particular IVR cycle (observed during each IVR insertion period except for IVR-4) and did not correlate with breakthrough bleeding or cycling except in ID23, which cleared LNG quickly after insertion of each IVR (Figs. 3, 4). LNG had no effect on the kinetics or levels of MIV-150 in blood or vaginal fluid (Fig. 2a–e), and LNG blood levels were unaffected by MZC (Fig. 2b–e).

We evaluated the effects of pore size (500 vs 800 μm), IVR composition (MZC vs MZCL), and repeated administration (IVR-1 vs IVR-5) on CG and MIV-150 PK. Pore size and IVR composition affected CG release from the IVR core. More CG was released through the 800 μm pore of MZCL IVRs (vaginal fluid level *p* < 0.0001, ratio = 0.03, 95% CI = 0.01–0.15) compared to the 500 μm pore. For IVRs with the smaller pore size, MZCL IVRs delivered less CG into the vaginal fluid than MZC IVRs (*p* = 0.0013, ratio = 13.27, 95% CI = 2.57–68.60; Fig. 2e). Repeated IVR administration influenced the PK of MIV-150 but not CG. For the MZCL IVRs, the total amount of MIV-150 detected in vivo varied between IVR-1 and IVR-5 (blood, *p* = 0.0004, ratio = 1.30, 95% CI = 1.12–1.51; vaginal fluid, *p* = 0.017, ratio = 3.94, 95% CI = 1.23–12.65), and this was independent of pore size (Fig. 2 **e**). For the MZCL and MZC IVRs, the total amount of CG detected in vivo varied by pore size but not IVR cycle (Fig. 2e). Regardless, sufficient CG was released, on average, to meet the target vaginal fluid concentration (>25 μg/ml).

**Fig. 5:**
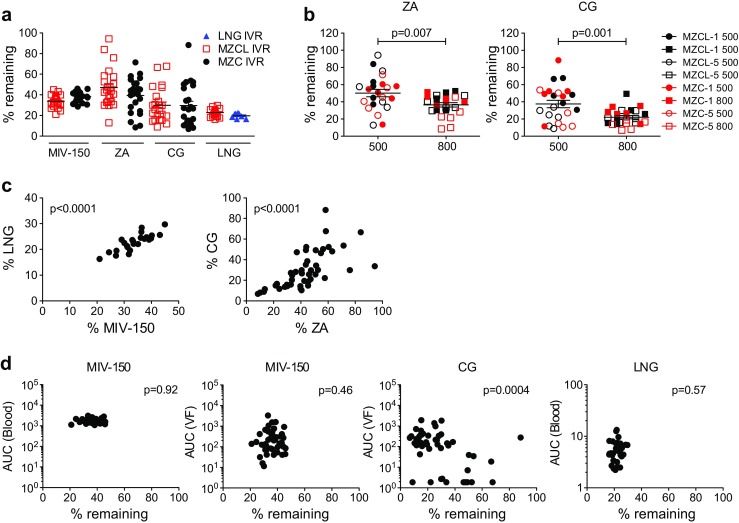
Residual drug levels in MZC and MZCL IVRs after removal. (**a**) The percentage of each drug remaining in the IVRs was determined by dividing drug content after removal by what was loaded. IVRs are shown individually from IVR-1 and IVR-5 cycles and both pore sizes from all macaques in each group. (**b**) The percentage of ZA and CG remaining in used IVRs by pore size and IVR cycle. *Closed symbols* represent IVR-1; *open symbols* represent IVR-5. (**c**) Spearman correlations between residual drug levels of the matrix (MIV-150 vs. LNG) and core (CG vs. ZA) components. (**d**) Correlations are shown between the total amounts of MIV-150, CG, and LNG detected in vivo and the amounts remaining in used IVRs post-removal

Release of all four drugs in vivo was verified by measuring residual drug levels in used (21d) IVRs. We examined only IVR-1 and IVR-5 because we had PK data from the same IVR cycles. Not more than 40% of MIV-150, 50% of ZA, 30% of CG, and 25% of LNG remained in the used IVRs on average (Fig. 5a). More ZA (*p* = 0.007, ratio = 1.761, 95% CI = 1.189–2.608) and CG (*p* = 0.001, ratio = 2.170, 95% CI = 1.418–3.321) remained in MZCL and MZC IVRs with a 500 μm pore compared to IVRs with an 800 μm pore, as expected, and residual CG and ZA levels were independent of the IVR cycle (Fig. 5b). Residual MIV-150 and LNG correlated (*p* < 0.0001, Spearman *r* = 0.8467, 95% CI = 0.666–0.933), as did residual ZA and CG (*p* < 0.0001, Spearman *r* = 0.776, 95% CI = 0.624–0.872; Fig. 5c). CG remaining in the IVRs correlated inversely with in vivo fluid levels (*p* = 0.0004, Spearman *r* = −0.493, 95% CI = −0.686–0.235; Fig. 5d). However, no correlation was seen for either MIV-150 or LNG (Fig. 5d).

### Efficacy of the MZCL IVR in macaques against repeated limiting-dose SHIV-RT/HSV-2 co-challenge

**Fig. 6:**
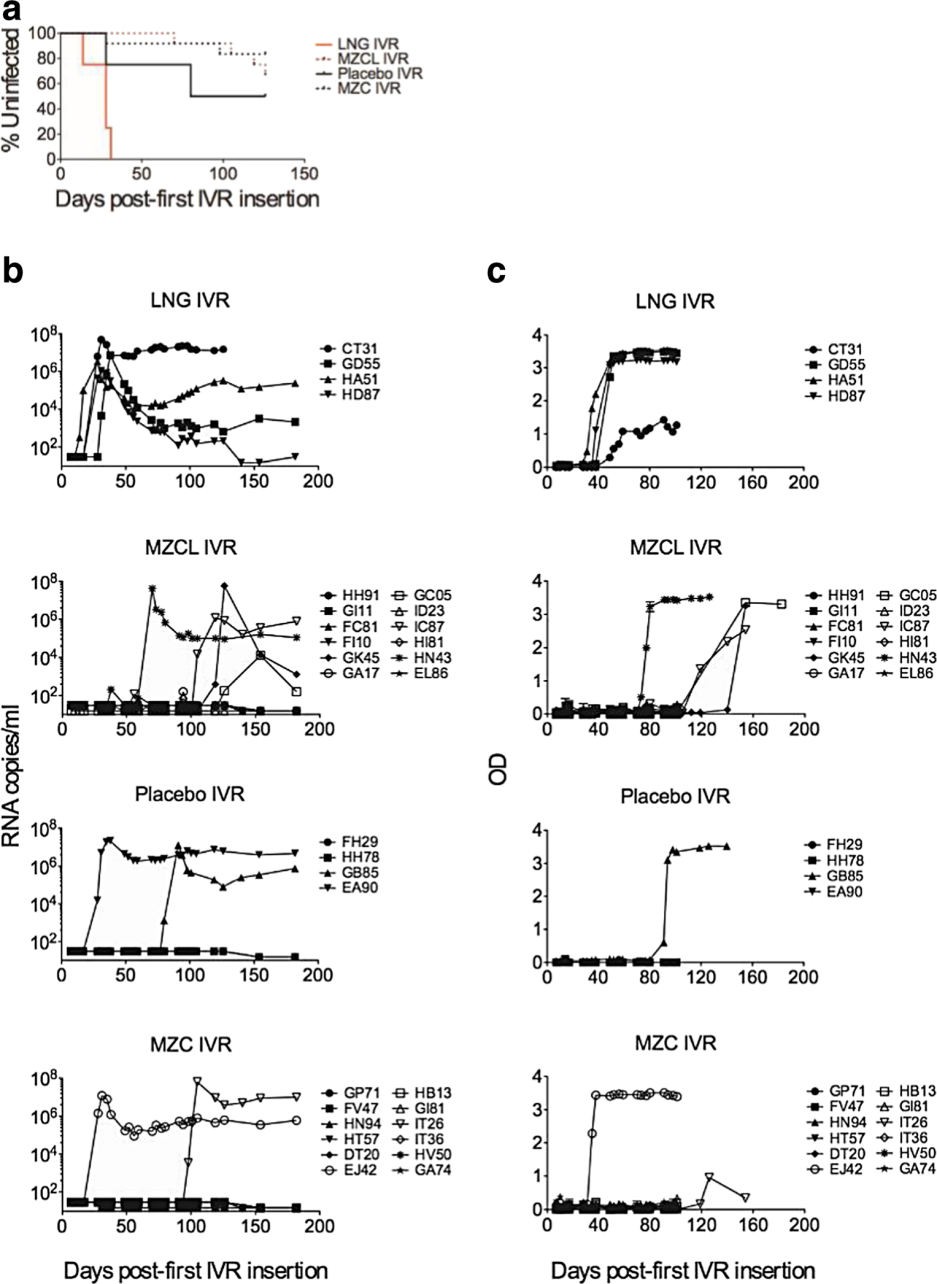
Anti-SHIV-RT activity of MZC and MZCL IVRs in macaques following repeated co-challenge with SHIV-RT and HSV-2. (**a**) The percent of macaques remaining SHIV negative over time was calculated by measuring SIV RNA in plasma collected immediately before each challenge (*n* = 4 LNG, *n* = 12 MZCL, *n* = 4 placebo, *n* = 12 MZC). (**b**) Plasma viral loads and (**c**) antibody development were monitored over time

The MZCL IVR significantly protected macaques against SHIV-RT acquisition (67% protection, log rank *p* = 0.046, Fig. 6a, b) in the context of repeated co-challenge with SHIV-RT and HSV-2 (see study schematic in Fig. 1c). Four of 12 macaques (33%) wearing MZCL IVRs became infected vs. four of four (100%) macaques wearing LNG IVRs (Table 1). The protection was more significant (67%, log rank *p* = 0.003) when comparing a combined MZC/MZCL group (6 of 24 infected, 25%) vs. an LNG/placebo control IVR group (6 of 8 infected, 75%), even though more macaques in the LNG vs. placebo IVR group became infected (4/4 vs. 2/4 infected; log rank *p* = 0.15) and macaques in the LNG group became infected earlier. The MZC and MZCL IVRs protected even when MIV-150 levels in vaginal fluid dropped below target (≥25 μg/ml) in some animals 14 d after IVR insertion (Fig. 2c). MZCL and MZC IVRs protected similarly (4 of 12 and 2 of 12, respectively, log rank *p* = 1.00). During IVR-1 and IVR-5, protection did not correlate with levels of CG and MIV-150 in vaginal fluid or systemic MIV-150 levels in individual macaques at the few PK time points tested. LNG serum levels, which were measured throughout all IVR cycles and at multiple time points (Fig. 3), also did not predict time of infection (Fig. 3 and Table 1). Infections in three of four macaques wearing MZCL IVRs were detected 1–4 wks after the IVR was removed. Macaque GC05 (MZCL IVR group) was the last macaque to become SHIV positive in plasma (at 4 wks post-last challenge) and exhibited a truncated viremia. Macaque CT31 (LNG IVR group) had to be euthanized 6 wks post-last challenge based on its sustained high SHIV plasma load and a poor SIV-specific antibody response. In all macaques except for GC05, plasma viral loads were identical regardless of treatment or time of infection (Fig. 6b). SIV-specific antibodies arose in all but one, EA90, of the infected macaques (Fig. 6c). EA90 was drug-naïve and became infected during an early challenge (first positive in plasma at challenge 5, Table 1).

Only one macaque, EJ42 from the MZC IVR group, had virus that contained an RT mutation: I178V (Table 2). EJ42 became infected early during the study and had mutant virus with I178V replicating in plasma during acute and chronic infection (100% of clones) as well as in PBMCs from chronic infection (91% of clones). I178V was not present in the inoculum (Table 2 and not shown). Although I178V does not arise preferentially with NNRTI use in humans [36], we previously found the I178V mutation in SHIV-RT-infected macaques under systemic MIV-150 pressure [32]. We examined the susceptibility of I178V-containing viruses to inhibition by MIV-150 or NNRTIs used in therapy like efavirenz, etravirine, and rilpivirine. SHIV-RT grown from co-cultures of EJ42 PBMCs with CX1 cells (100% I178V, Table 2) and HIV_NL43_ engineered by site-directed mutagenesis with I178V both remained susceptible to the four NNRTIs, which indicates that I178V does not confer resistance to those NNRTIs (Table 3).

**Table 2:** Screening for NNRTI-resistance-associated mutations in macaques infected with SHIV-RT in the presence of IVRs

IVR	Animal ID	Time point tested	L100, K101, K103, V108, E138, I178, V179, Y181, Y188, G190, P225
MZCL	GK45	Wk 8, Wk 12	NA^a^
	IC87	Wk 6	0 (23)^b^
	HN43	Wk 2	0 (24)
	GC05	Wk 8	NA
MZC	EJ42	Plasma: Ch 5–8, Wk 2, Wk 30 PBMC: Wk 30	Plasma: 67 (68) I178V^c^ PBMC: 20 (23) I178V 1 (23) I178V + K101R Cultured PBMC: 19 (19) I178V
	IT26	Wk 6	0 (20)
		Stock SHIV-RT	0 (32)

^a^NA indicates that no RNA could be isolated from available plasma samples, likely due to low viral load and/or volume of plasma

^b^The number of clones in which amino acid mutations conferring NNRTI resistance were detected. Parentheses indicate the total number of clones sequenced

^c^I178V was present in 23/23 clones of plasma virus from acute infection (pooled plasma from challenge 5–8) and 44/45 clones from chronic infection (23/23 at Wk 2 post-last challenge and 21/22 at Wk 30 post-last challenge)

**Table 3:** NNRTI-resistance profile of I178V

NNRTI	EC_50_ (95% confidence interval)			
	SHIV-RT WT	SHIV-RT_EJ42_ I178V	HIV-1_NL4-3_ WT	HIV-1_NL4-3_ I178V
MIV-150	1.1 (0.9–1.3)	1.1 (0.8–1.5)	0.96 (0.80–1.14)	0.70 (0.61–0.82)
Efavirenz	ND	ND	1.22 (1.05–1.42)	0.80 (0.66–0.96)
Etravirine	3.1 (2.5–3.8)	2.2 (1.3–3.5)	0.54 (0.42–0.68)	0.23 (0.18–0.29)
Rilpivirine	0.5 (0.4–0.7)	0.3 (0.2–0.4)	1.79 (1.48–2.16)	0.92 (0.70–1.21)

*ND* not determined

**Fig. 7:**
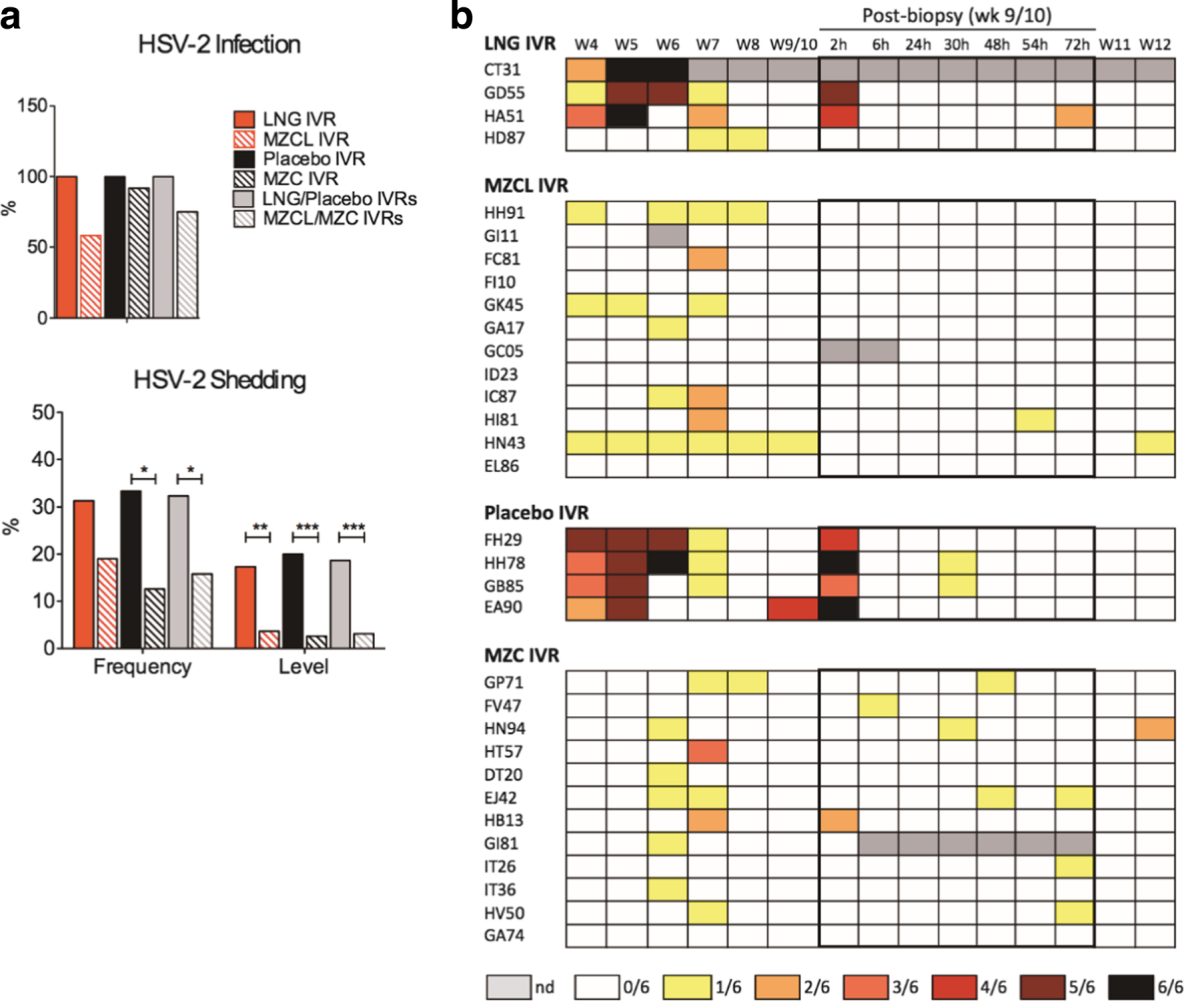
Anti-HSV-2 activity of MZC and MZCL IVRs in macaques following repeated co-challenge with SHIV-RT and HSV-2. (**a**) HSV-2 infection and vaginal shedding were assessed by nested PCR in gD on vaginal fluid and cervical and vaginal tissue biopsies (*n* = 4 LNG, *n* = 12 MZCL, *n* = 4 placebo, *n* = 12 MZC). Evaluation commenced 4 wks after the last challenge. (**b**) Heat map depicting HSV-2 shedding in vaginal fluid observed over time for LNG, MZCL, placebo, and MZC groups from (**a**). *Each row* represents an animal. Shedding was measured at wks 4, 5, 6, 7, 8, and 9, or 10, and then again at 2, 6, 24, 30, 48, 54, and 72 h following biopsy. The legend shows the colors representing the fraction of replicate PCR reactions positive of 6 total with *gray* indicating not determined (nd), representing that no sample was available at that time point

MZC and MZCL IVRs decreased HSV-2 infection but not significantly (Fig. 7a). However, they did significantly reduce vaginal fluid HSV-2 shedding frequency (*p* = 0.02, OR = 0.38, 95% CI = 0.16–0.88) and the levels of HSV-2 DNA detected at shedding time points (*p* < 0.0001, OR = 0.14, 95% CI = 0.06–0.31) (Fig. 7a,b), which could result in decreased infectivity of the vaginal fluid of these animals. MZCL and MZC IVRs also reduced HSV-2 shedding levels compared to the LNG and placebo controls, respectively (MZCL *p* = 0.0029, OR = 0.18, 95% CI = 0.06–0.59; MZC *p* < 0.0001, OR = 0.10, 95% CI = 0.03–0.31).

### Effect of the menstrual cycle on SHIV-RT infection

**Fig. 8:**
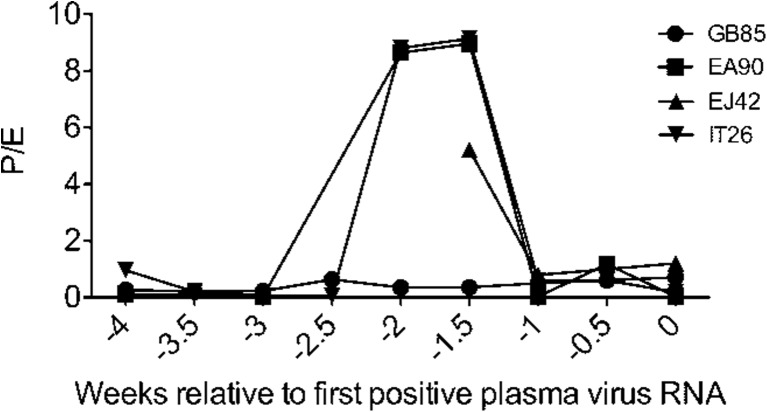
SHIV infection during luteal phase of the menstrual cycle. The ratio of progesterone (P) to estradiol (E) was calculated for animals not exposed to LNG that became infected during the study. P/E for each animal was overlaid beginning 4 wks before plasma virus RNA detection (−4)

Serum progesterone and estradiol levels fluctuated (Fig. 4), allowing us to calculate the number of menstrual cycles (mean ± SEM) over the entire 137-d study as follows: 3.75 ± 0.63 for the placebo group, 3.42 ± 0.23 for MZC, 1.00 ± 0.41 for LNG, and 1.42 ± 0.45 for MZCL. These data indicate that LNG-containing IVRs released sufficient LNG to suppress cycling (*p* = 0.04, LNG [mean ± SEM = 1.00 ± 0.41] vs. placebo [mean ± SEM = 3.75 ± 0.63]), and this was not affected by MZC (*p* = 0.95, MZCL [mean ± SEM = 1.42 ± 0.45] vs. LNG [mean ± SEM = 1.00 ± 0.41]). The luteal phase was defined by a progesterone level above 1 ng/ml [37]. And notably, three of the four animals that became infected with SHIV in the absence of LNG were infected during late luteal phase, the time of the menstrual cycle when women (and also cycling rhesus and pigtail macaques) have estrogen/progesterone levels favorable for HIV infection and are the most susceptible to HIV infection [20, 38–45] (Fig. 8).

### Effect of biological fluids on antiviral activity

**Fig. 9:**
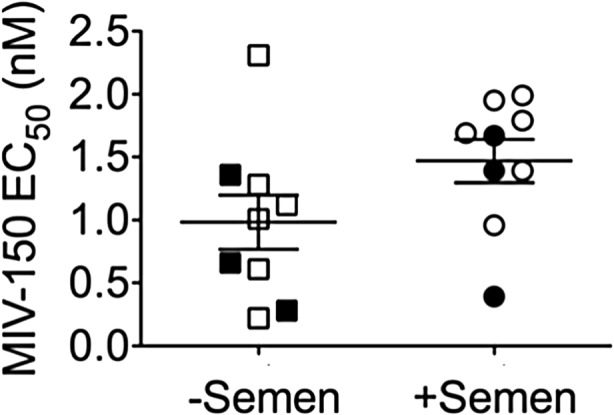
Effect of biological fluids on the antiviral properties of the MZC combination. The effect of semen (Lee Biosciences) on the in vitro anti-HIV activity of MZC released in vivo from MZCL (closed symbols, *n* = 3) and MZC (open symbols, *n* = 6) IVRs was measured in macaque vaginal fluid. The EC_50_ values were estimated using the TZM-bl assay

The presence of semen neither increased nor decreased the anti-HIV activity of vaginal fluid from macaques treated with MZCL/MZC IVRs (*p* = 0.066, ratio = 0.133, 95% CI = 0.018–0.978, Fig. 9); EC_50_ values matched that of native MIV-150 (Table 3). Previously, we showed that vaginal fluid and semen do not interfere with CG’s anti-HPV activities in vitro [17, 23].

## Discussion

MPT IVR development requires a multipronged approach that aims to correlate in vivo biological effects in animal models with PK/PD and with IVR performance measures like in vitro and in vivo release profiles. This approach becomes even more challenging when the MPT contains drugs that target multiple indications, when analytical methods to detect drugs in all biological matrices are not validated, when absorption of the drugs differs, and when a single animal model to fully evaluate multiple biological outcomes is unavailable or unfeasible. To overcome these challenges, we used a variety of methods to correlate IVR performance and PK with biological effects.

Herein, we have demonstrated that a prototype MZCL IVR released sufficient quantities of its drug payload to significantly reduce SHIV-RT infection and HSV-2 vaginal shedding in macaques exposed to repeated SHIV-RT/HSV-2 co-challenges and shut down hormonal cycling. The study utilized a modified version of our published repeated SHIV-RT/HSV-2 co-challenge model. For proof-of-concept testing of the prototype IVRs against both viruses, we modified the schedule (challenge 4 times in 10 days followed by 11 days of no challenge) so as to challenge macaques with SHIV-RT and HSV-2 during a time period in which the animals would most likely be protected based on drug concentrations. An optimized IVR with stabilized release profile will need to be tested in a standard challenge regimen (e.g., weekly exposure) as has been used by other studies of IVR efficacy [46–48]. Importantly, the MZCL IVR protected even in the context of 20 twice-weekly co-exposures in contrast to other IVR efficacy studies using SHIV alone in a weekly challenge regimen often with fewer challenges [46–48]. The in vivo antiviral activity for the MZCL IVR matches our data on MZC gel in a similar repeated co-challenge model [20] and also matches efficacy data on an MIV-150 IVR that released the drug with similar pharmacokinetics in vivo [23, 30]. We have previously found that MZC gel significantly protects mice against HPV pseudovirus (PsV) infection [10]. In addition, CG reduced HPV16 PsV infection in macaques [16] and a recent study of women who used the CG-based lubricant Divine 9 found that cervicovaginal lavages containing more than 0.4 μg/ml CG exhibited more than 95% inhibition of HPV16 PsV infection in vitro [29]. Based on CG levels seen in vaginal fluid in this study, we expect that the MZCL IVR will also reduce HPV infection significantly [10, 23], although we were unable to directly evaluate that herein.

MZC and MZCL IVRs significantly protected macaques against SHIV-RT even in the absence of 100% infection frequency in the controls, a common result in limiting dose vaginal challenge studies in rhesus macaques in the absence of hormonal treatment (i.e., DMPA [20, 49–51]). Plasma viremia was delayed in three of four animals from the MZCL group and zero of two animals in the MZC group, detected only after IVR-5 was removed. This suggests that those animals likely became infected on the last or close to the last challenge and had a low-level infection that only became detectable systemically weeks after infection. There are several possible reasons why animals in the MZCL IVR group, in particular, exhibited delayed viremia. Because we were not able to look at many tissue sites in this study, we cannot rule out there may have been low levels of virus replicating in distal tissues that ultimately gave rise to systemic infection. It is possible that effects of LNG (delivered by the MZCL IVR) in the vaginal mucosa allowed low level infections to take hold in this group but not in the MZC IVR group. It is also possible that residual systemic or tissue MIV-150 levels may have played a role in controlling a low level infection until the drug was completely cleared from the tissues. In support of these possibilities, we have previously shown that a MIV-150 IVR protected macaques only when it was in place post-challenge [30]. In this case, had we left the IVRs in place for longer than 4 d after the last challenge, we might have seen better protection by the MZCL IVRs. That the macaques became more readily infected during IVR-5 vs. IVR-1 could also possibly reflect the lower levels of MIV-150 detected overall in vivo from MZCL IVR-5 vs. IVR-1. Both plasma and vaginal fluid MIV-150 levels were consistently lower during MZCL IVR-5 compared to IVR-1 despite similar amounts of MIV-150 having been released from the IVRs (as determined by residual levels of MIV-150 in the used IVRs). Quality control testing showed similar drug loading in the two batches of IVRs, suggesting that batch-to-batch variability was not the cause. Lower levels of MIV-150 in vaginal tissues and/or shorter residence time there could have contributed to the SHIV-RT infections that occurred post-removal of MZCL IVR-5. In prior studies, we found that the best correlate of protection from SHIV-RT infection was concentration of MIV-150 in genital tissue [19, 30], which we were unable to measure herein, since sampling of vaginal tissue at or around the time of virus challenge would have perturbed the mucosal environment and perhaps increased infection frequencies. Prolonged exposure to MIV-150 could have led to increased levels of MIV-150-metabolizing enzymes, as seen with other NNRTIs [52]. We have also observed that repeated intramuscular dosing of MIV-150 results in lower peak levels post-injection over time [32]. Finally, since we co-challenged with HSV-2 and SHIV-RT, it is possible that the lag time to systemic infection may be related to the inflammatory environment created by HSV-2 co-exposure (regardless of eventual HSV-2 infection). We did not have the samples to check if these animals in the MZCL group that became SHIV+ weeks after the last challenge had a greater concentration of inflammatory mediators in their vaginal fluid or a heightened inflammatory state systemically.

LNG could have contributed to the observed loss of protection in MZCL IVR-carrying macaques post-IVR-5 removal. Although the study was not designed or powered to assess LNG effects on the vaginal microenvironment, LNG released from the IVRs might have made the vaginal epithelium more permissive to infection or increased infection frequency through another mechanism. In turn, this could have increased the requirement for post-challenge MIV-150 coverage, resulting in decreased protection after removal of IVR-5. However, LNG levels in vivo were not associated with time of infection. The impact of LNG should be explored further given the widespread use of LNG-containing contraceptives [22] and development of LNG-containing MPTs [3]. Epidemiological studies suggest that some forms of hormonal contraception might increase HIV transmission and acquisition [53–55], but the data so far remain inconclusive, and more studies, such as the ongoing ECHO trial, are needed to fully understand the relationship between hormones and HIV transmission [56]. Recent microarray analyses of cervical and endometrial tissues taken from women using DMPA or the LNG intrauterine system revealed that progestins influence the expression of immune-related genes that could alter susceptibility to HIV infection and that this was particularly apparent in the endometrial tissues [57]. However, no data are available on the risk of HIV acquisition when hormones are delivered in low doses via an IVR, and additional studies designed specifically to address this are needed. Critically, LNG did not reduce the overall efficacy of continuously dosed MZC microbicide.

The MZCL IVR significantly reduced HSV-2 shedding in infected macaques but not HSV-2 infection outright. The reduced shedding could reflect a blunted infection in the mucosa, less robust neural infection, and/or control of virus by mucosal innate immune mediators that were triggered by sustained release of ZA and CG from the IVRs [58–65]. However, we did not collect data on HSV-2 in dorsal root ganglia (via necropsy) and immune mediators in vaginal fluid. We have previously found that macaques that became infected after receiving MZC gel vaginally did not develop an HSV-2-specific T cell response [13], suggesting that adaptive immunity did not play a role in the reduced shedding. The inability of the MZCL IVR to protect macaques from infection may be related to the high dose of HSV-2 used in the co-challenge model. A large body of data exists for SHIV doses in the macaque, but there are no data on optimal, relevant doses for HSV-2. The HSV-2 dose in this study is based on our single high-dose co-challenge model [13, 20, 66]. We administered the same total inoculum (2 × 10^8^ pfu) spread over 20 challenges (10^7^ pfu per challenge). This high dose of HSV-2 inoculum ensures 100% HSV-2 infection in the control groups; however, it is 3–5 logs higher than that found in infected people [67–69] and may have overwhelmed the ZC-mediated protection. In mice, protection from HSV-2 depends on the HSV-2 challenge dose [10, 12, 14]. Lowering the HSV-2 inoculum and/or optimizing ZC release/content may improve MZC’s anti-HSV-2 activity and reduce HSV-2 shedding further. ZA and CG release (PK and residual drug levels in IVRs) was more variable overall than MIV-150 and LNG release. ZA and CG were both released from the core and may have been more affected by inter-animal differences in vaginal fluid volume and viscosity. As evident in Table 1, ZA/CG released through neither the 500 nor 800 μm pore-inhibited HSV-2 infection. Importantly, reduced viral shedding in HSV-2-infected individuals could reduce subsequent transmission of HSV-2 to partners. And since HSV-2 infection increases the likelihood of HIV acquisition in humans and SHIV acquisition in macaques, reduced viral shedding might potentially reduce the acquisition or transmission of HIV [66, 70].

There is no established macaque model to simultaneously evaluate the efficacy of an MPT against HIV, HSV-2, and HPV, so surrogate markers of in vivo efficacy are typically used. In our initial report, we showed that vaginal fluid from macaques treated with the MZCL IVR prevented HPV infection in vitro [23]. As in that study, in vivo CG levels herein were ∼1000 times the in vitro EC_50_ for CG [10, 17, 71] and sufficient to block HPV pseudovirus infection in mice [10, 15, 17].

Rhesus macaques have irregular seasonal cycles and are not the ideal subspecies for modeling women’s menstrual cycles [31]. By contrast, pigtail macaques cycle monthly throughout the year, and studies evaluating the role of female sex hormone fluctuations on HIV acquisition have used the pigtail macaque model [72]. Pigtail macaques are harder to obtain than rhesus. We have also shown that we can detect the menstrual cycle of some rhesus macaques [20, 73]. For those reasons, we use rhesus macaques as our model species. In the current study, we clearly demonstrated that rhesus macaques were cycling during the ovulatory season, also showing the impact of LNG-releasing IVRs on cycling as a correlate of contraceptive effect.

PK and cumulative IVR release data were collected to inform on the efficacy data and support the preclinical/clinical development of a human-sized MZCL IVR. These data enabled us to identify drug–drug interactions or combinations that influenced release of each drug, validate in vitro and initial in vivo release data, and identify potential design improvements. An important caveat is that in vivo release characteristics of the IVRs in the prior study did not parallel the in vitro release kinetics and overall profile [23]. Based on the drug levels remaining in the IVRs, we concluded that the amount of each drug released in vivo exceeds estimates derived from in vitro data. We are working to identify the in vitro release condition that best mimics the in vivo profile, and it will be used to characterize optimized IVRs before they are tested in vivo.

As expected, in vivo release of the hydrophilic core components ZA and CG but not the hydrophobic matrix components MIV-150 and LNG was driven predominantly by pore size [23]. However, in IVRs with the smaller 500 μm pore, LNG reduced CG (and presumably ZA) release significantly. This could potentially reflect LNG-driven changes in cervical mucus [74] that may have impeded hydration of the core through the smaller pore and/or the spread of hydrated core components away from the smaller pore opening. Correlation between drug levels in plasma and vaginal fluid and cumulative release for CG but not MIV-150 or LNG is consistent with CG being retained in the vaginal lumen and MIV-150 and LNG being possibly accumulated in tissues, absorbed into the systemic circulation, and eliminated. Despite having different in vivo ZC release profiles, the 500 and 800 μm pore IVRs effectively prevented SHIV-RT infection and HSV-2 shedding. Although ZA levels in vaginal fluid could not be measured in vivo due to lack of a validated analytical method, we verified its release by quantifying the residual drug levels in the IVRs after removal. As predicted from in vitro release studies [23], overall ZA release mirrored overall CG release in vivo. The consistent release of ZC from the MZCL IVRs likely contributed to the significant reduction in HSV-2 shedding and may also have contributed to the IVR’s anti-SHIV efficacy, as was seen for MZC gel [10, 19]. However, we were unable to include the additional IVR groups needed to test this hypothesis in the current study. And because we could not identify the time of HSV-2 infection, HSV-2 infection outcomes and CG (and ZA) levels in vaginal fluid could not be correlated. Thus, we were also unable to assess if the MZC-containing IVRs increased the number of challenges needed to result in HSV-2 infection.

Knowing semen’s potential for enhancing HIV infection [75] and the suggested link between semen effects and failure of microbicides in clinical trials [76], we evaluated the effect of semen on the activity of the antiviral drugs released in vivo from the MZC and MZCL IVRs. Here, we found that the anti-HIV activity of vaginal fluid from MZCL/MZC IVR-treated macaques was unaffected by semen. This is in line with our previous report on MZC gel in vaginal explants treated with seminal plasma [77]. Other experiments not reported here suggest that biological fluids do not interfere with the anti-HSV-2 properties of the ZC combination (unpublished). Seminal plasma actually increased the survival of HEC-treated mice in the HSV-2 model compared to HEC alone, consistent with what others had observed [78]. While we were unable to measure the impact of semen or seminal plasma on the anti-HPV activity of CG released from MZCL IVRs in vivo, CG’s anti-HPV activity was previously confirmed in mice co-exposed with seminal plasma [17].

Topically applied microbicides containing antiretroviral drugs may induce the development of drug-resistant virus. Screening for drug-resistance mutations in drug-treated animals that become infected in preclinical studies gauges the likelihood of drug resistance emerging in clinical settings. The I178V mutation that arose in plasma and PBMC virus from EJ42 neither resulted in a divergent profile of SHIV plasma viremia or HSV-2 shedding compared to other animals nor reduced the susceptibility of either SHIV-RT or HIV to NNRTI-mediated inhibition. One clone of the PBMC virus contained the combination of I178V and K101R. K101E, K101P, and K101H can reduce susceptibility to NNRTIs [36], and K101 is an important amino acid in the interaction of MIV-150 with the hydrophobic pocket of RT [79]. However, K and R both have a positively charged side chain that provides the H-bonding necessary for MIV-150 to interact with RT. While the presence of these mutations indicates the influence of drug pressure on SHIV-RT in this animal, the concentrations of MIV-150 achieved in vivo did not support the establishment of NNRTI-resistant virus.

Unlike other MPTs in development, the MZCL IVR is designed to simultaneously prevent HIV, HSV-2, HPV, and unintended pregnancy. The data presented herein supports that notion. MZC delivered from the IVR retained its potent antiviral activity in the presence of vaginal fluid and seminal components, significantly preventing SHIV infection and reducing HSV-2 shedding. Its anti-HPV activity was inferred from CG levels in vaginal fluid. LNG delivered from the IVR suppressed cycling in rhesus macaques. By integrating PK, IVR release, and efficacy data into one model, we can identify the IVR’s critical performance parameters and inform development of the optimized MZCL IVR, a product that has the potential to safely and effectively protect millions of women worldwide from three incurable viral infections and unintended pregnancy.

## References

[1] Schelar E, Polis CB, Essam T, Looker KJ, Bruni L, Chrisman CJ (2016). Multipurpose prevention technologies for sexual and reproductive health: mapping global needs for introduction of new preventive products. Contraception.

[2] Fernandez-Romero JA, Teleshova N, Zydowsky TM, Robbiani M (2015). Preclinical assessments of vaginal microbicide candidate safety and efficacy. Adv Drug Deliv Rev.

[3] Fernandez-Romero JA, Deal C, Herold BC, Schiller J, Patton D, Zydowsky T (2015). Multipurpose prevention technologies: the future of HIV and STI protection. Trends Microbiol.

[4] Marrazzo JM, Ramjee G, Richardson BA, Gomez K, Mgodi N, Nair G (2015). Tenofovir-based preexposure prophylaxis for HIV infection among African women. N Engl J Med.

[5] Rees H, Delany-Moretlwe S, Lombard C, Baron D, Panchia R, Myer L et al. FACTS 001 Phase III trial of pericoital tenofovir 1% gel for HIV prevention in women. CROI 2015; Seattle, WA2015.

[6] Dai JY, Hendrix CW, Richardson BA, Kelly C, Marzinke M, Chirenje ZM (2016). Pharmacological measures of treatment adherence and risk of HIV infection in the VOICE study. J Infect Dis.

[7] Baeten JM, Palanee-Phillips T, Brown ER, Schwartz K, Soto-Torres LE, Govender V, et al. Use of a vaginal ring containing dapivirine for HIV-1 prevention in women. N Engl J Med. 2016; doi:10.1056/NEJMoa1506110. 10.1056/NEJMoa1506110 PMC4993693 26900902

[8] Derby N, Zydowsky T, Robbiani M (2013). In search of the optimal delivery method for anti-HIV microbicides: are intravaginal rings the way forward?. Expert Rev Anti-Infect Ther.

[9] Friend DR, Kiser PF (2013). Assessment of topical microbicides to prevent HIV-1 transmission: concepts, testing, lessons learned. Antivir Res.

[10] Kizima L, Rodriguez A, Kenney J, Derby N, Mizenina O, Menon R (2014). A potent combination microbicide that targets SHIV-RT, HSV-2 and HPV. PLoS One.

[11] Fenstermacher KJ, DeStefano JJ (2011). Mechanism of HIV reverse transcriptase inhibition by zinc: formation of a highly stable enzyme-(primer-template) complex with profoundly diminished catalytic activity. J Biol Chem.

[12] Fernandez-Romero JA, Abraham CJ, Rodriguez A, Kizima L, Jean-Pierre N, Menon R (2012). Zinc acetate/carrageenan gels exhibit potent activity in vivo against high-dose herpes simplex virus 2 vaginal and rectal challenge. Antimicrob Agents Chemother.

[13] Hsu M, Aravantinou M, Menon R, Seidor S, Goldman D, Kenney J (2014). A combination microbicide gel protects macaques against vaginal simian human immunodeficiency virus-reverse transcriptase infection, but only partially reduces herpes simplex virus-2 infection after a single high-dose cochallenge. AIDS Res Hum Retrovir.

[14] Kenney J, Rodriguez A, Kizima L, Seidor S, Menon R, Jean-Pierre N (2013). A modified zinc acetate gel, a potential nonantiretroviral microbicide, is safe and effective against simian-human immunodeficiency virus and herpes simplex virus 2 infection in vivo. Antimicrob Agents Chemother.

[15] Roberts JN, Buck CB, Thompson CD, Kines R, Bernardo M, Choyke PL (2007). Genital transmission of HPV in a mouse model is potentiated by nonoxynol-9 and inhibited by carrageenan. Nat Med.

[16] Roberts JN, Kines RC, Katki HA, Lowy DR, Schiller JT (2011). Effect of Pap smear collection and carrageenan on cervicovaginal human papillomavirus-16 infection in a rhesus macaque model. J Natl Cancer Inst.

[17] Rodriguez A, Kleinbeck K, Mizenina O, Kizima L, Levendosky K, Jean-Pierre N (2014). In vitro and in vivo evaluation of two carrageenan-based formulations to prevent HPV acquisition. Antivir Res.

[18] Marais D, Gawarecki D, Allan B, Ahmed K, Altini L, Cassim N (2011). The effectiveness of Carraguard, a vaginal microbicide, in protecting women against high-risk human papillomavirus infection. Antivir Ther.

[19] Kenney J, Aravantinou M, Singer R, Hsu M, Rodriguez A, Kizima L (2011). An antiretroviral/zinc combination gel provides 24 hours of complete protection against vaginal SHIV infection in macaques. PLoS One.

[20] Kenney J, Derby N, Aravantinou M, Kleinbeck K, Frank I, Gettie A (2014). Short communication: a repeated simian human immunodeficiency virus reverse transcriptase/herpes simplex virus type 2 cochallenge macaque model for the evaluation of microbicides. AIDS Res Hum Retrovir.

[21] Kenney J, Singer R, Derby N, Aravantinou M, Abraham CJ, Menon R (2012). A single dose of a MIV-150/zinc acetate gel provides 24 h of protection against vaginal simian human immunodeficiency virus reverse transcriptase infection, with more limited protection rectally 8–24 h after gel use. AIDS Res Hum Retrovir.

[22] WHO. List of essential medicines. 2013. http://www.who.int/medicines/publications/essentialmedicines/18th_EML_Final_web_8Jul13.pdf.

[23] Ugaonkar SR, Wesenberg A, Wilk J, Seidor S, Mizenina O, Kizima L (2015). A novel intravaginal ring to prevent HIV-1, HSV-2, HPV, and unintended pregnancy. J Control Release.

[24] Begay O, Jean-Pierre N, Abraham CJ, Chudolij A, Seidor S, Rodriguez A (2011). Identification of personal lubricants that can cause rectal epithelial cell damage and enhance HIV type 1 replication in vitro. AIDS Res Hum Retrovir.

[25] Trkola A, Matthews J, Gordon C, Ketas T, Moore JP (1999). A cell line-based neutralization assay for primary human immunodeficiency virus type 1 isolates that use either the CCR5 or the CXCR4 coreceptor. J Virol.

[26] Ashley R, Schmidt N, Emmons R (1995). Herpes simplex viruses. Diagnostic procedures for viral, rickettsial, and chlamydial infections.

[27] Animal Welfare Act and Regulation Code of Federal Regulations. Beltsville, MD: Department of Agriculture; 2001.

[28] Guide for the Care and Use of Laboratory Animals. The National Academies Press; 2011. 21595115

[29] Novetsky AP, Keller MJ, Gradissimo A, Chen Z, Morgan SL, Xue X (2016). In vitro inhibition of human papillomavirus following use of a carrageenan-containing vaginal gel. Gynecol Oncol.

[30] Singer R, Mawson P, Derby N, Rodriguez A, Kizima L, Menon R (2012). An intravaginal ring that releases the NNRTI MIV-150 reduces SHIV transmission in macaques. Sci Transl Med.

[31] Jensen JT, Zelinski MB, Stanley JE, Fanton JW, Stouffer RL (2008). The phosphodiesterase 3 inhibitor ORG 9935 inhibits oocyte maturation in the naturally selected dominant follicle in rhesus macaques. Contraception.

[32] Hsu M, Keele BF, Aravantinou M, Krawczyk N, Seidor S, Abraham CJ (2014). Exposure to MIV-150 from a high-dose intravaginal ring results in limited emergence of drug resistance mutations in SHIV-RT infected rhesus macaques. PLoS One.

[33] Luukkainen T, Toivonen J (1995). Levonorgestrel-releasing IUD as a method of contraception with therapeutic properties. Contraception.

[34] Nilsson CG, Lahteenmaki P, Luukkainen T (1980). Levonorgestrel plasma concentrations and hormone profiles after insertion and after one year of treatment with a levonorgestrel-IUD. Contraception.

[35] Xiao BL, Zhang XL, Feng DD (1985). Pharmacokinetic and pharmacodynamic studies of vaginal rings releasing low-dose levonorgestrel. Contraception.

[36] Stanford University HIV Drug resistance database. 2016.

[37] Walker ML, Gordon TP, Wilson ME (1983). Menstrual cycle characteristics of seasonally breeding rhesus monkeys. Biol Reprod.

[38] Hadzic SV, Wang X, Dufour J, Doyle L, Marx PA, Lackner AA (2014). Comparison of the vaginal environment of *Macaca mulatta* and *Macaca nemestrina* throughout the menstrual cycle. Am J Reprod Immunol.

[39] Hel Z, Stringer E, Mestecky J (2010). Sex steroid hormones, hormonal contraception, and the immunobiology of human immunodeficiency virus-1 infection. Endocr Rev.

[40] Hild-Petito S, Veazey RS, Larner JM, Reel JR, Blye RP (1998). Effects of two progestin-only contraceptives, Depo-Provera and Norplant-II, on the vaginal epithelium of rhesus monkeys. AIDS Res Hum Retrovir.

[41] Marx PA, Spira AI, Gettie A, Dailey PJ, Veazey RS, Lackner AA (1996). Progesterone implants enhance SIV vaginal transmission and early virus load. Nat Med.

[42] McNicholl JM, Henning TC, Vishwanathan SA, Kersh EN (2014). Non-human primate models of hormonal contraception and HIV. Am J Reprod Immunol.

[43] Nichols WA, Birke L, Dufour J, Loganantharaj N, Bagby GJ, Nelson S (2015). Characterization of the genital microenvironment of female rhesus macaques prior to and after SIV infection. Am J Reprod Immunol.

[44] Smith SM, Baskin GB, Marx PA (2000). Estrogen protects against vaginal transmission of simian immunodeficiency virus. J Infect Dis.

[45] Sodora DL, Gettie A, Miller CJ, Marx PA (1998). Vaginal transmission of SIV: assessing infectivity and hormonal influences in macaques inoculated with cell-free and cell-associated viral stocks. AIDS Res Hum Retrovir.

[46] Fetherston SM, Geer L, Veazey RS, Goldman L, Murphy DJ, Ketas TJ (2013). Partial protection against multiple RT-SHIV162P3 vaginal challenge of rhesus macaques by a silicone elastomer vaginal ring releasing the NNRTI MC1220. J Antimicrob Chemother.

[47] Smith JM, Rastogi R, Teller RS, Srinivasan P, Mesquita PM, Nagaraja U (2013). Intravaginal ring eluting tenofovir disoproxil fumarate completely protects macaques from multiple vaginal simian-HIV challenges. Proc Natl Acad Sci U S A.

[48] Srinivasan P, Moss JA, Gunawardana M, Churchman SA, Yang F, Dinh CT (2016). Topical delivery of tenofovir disoproxil fumarate and emtricitabine from pod-intravaginal rings protects macaques from multiple SHIV exposures. PLoS One.

[49] Arrode-Bruses G, Goode D, Kleinbeck K, Wilk J, Frank I, Byrareddy S (2016). A small molecule, which competes with MAdCAM-1, activates integrin alpha4beta7 and fails to prevent mucosal transmission of SHIV-SF162P3. PLoS Pathog.

[50] Cheng-Mayer C, Huang Y, Gettie A, Tsai L, Ren W, Shakirzyanova M (2011). Delay of simian human immunodeficiency virus infection and control of viral replication in vaccinated macaques challenged in the presence of a topical microbicide. AIDS.

[51] Goode D, Truong R, Villegas G, Calenda G, Guerra-Perez N, Piatak M (2014). HSV-2-driven increase in the expression of alpha4beta7 correlates with increased susceptibility to vaginal SHIV(SF162P3) infection. PLoS Pathog.

[52] Weiss J, Haefeli WE (2013). Potential of the novel antiretroviral drug rilpivirine to modulate the expression and function of drug transporters and drug-metabolising enzymes in vitro. Int J Antimicrob Agents.

[53] Haddad LB, Philpott-Jones S, Schonfeld T (2015). Contraception and prevention of HIV transmission: a potential conflict of public health principles. J Fam Plann Reprod Health Care.

[54] Morrison CS, Chen PL, Kwok C, Baeten JM, Brown J, Crook AM (2015). Hormonal contraception and the risk of HIV acquisition: an individual participant data meta-analysis. PLoS Med.

[55] Ralph LJ, McCoy SI, Shiu K, Padian NS (2015). Hormonal contraceptive use and women’s risk of HIV acquisition: a meta-analysis of observational studies. Lancet Infect Dis.

[56] Westhoff CL, Winikoff B (2014). DMPA and HIV: do we need a trial?. Contraception.

[57] Goldfien GA, Barragan F, Chen J, Takeda M, Irwin JC, Perry J (2015). Progestin-containing contraceptives alter expression of host defense-related genes of the endometrium and cervix. Reprod Sci.

[58] Hirano T, Murakami M, Fukada T, Nishida K, Yamasaki S, Suzuki T (2008). Roles of zinc and zinc signaling in immunity: zinc as an intracellular signaling molecule. Adv Immunol.

[59] Kahmann L, Uciechowski P, Warmuth S, Plumakers B, Gressner AM, Malavolta M (2008). Zinc supplementation in the elderly reduces spontaneous inflammatory cytokine release and restores T cell functions. Rejuvenation Res.

[60] Mocchegiani E, Giacconi R, Muzzioli M, Cipriano C (2000). Zinc, infections and immunosenescence. Mech Ageing Dev.

[61] Mocchegiani E, Giacconi R, Muzzioli M, Gasparini N, Provinciali L, Spazzafumo L (2000). Different age-related effects of thymectomy in myasthenia gravis: role of thymoma, zinc, thymulin, IL-2 and IL-6. Mech Ageing Dev.

[62] Mocchegiani E, Muzzioli M (2000). Therapeutic application of zinc in human immunodeficiency virus against opportunistic infections. J Nutr.

[63] Mocchegiani E, Muzzioli M, Giacconi R (2000). Zinc, metallothioneins, immune responses, survival and ageing. Biogerontology.

[64] Rink L, Kirchner H (2000). Zinc-altered immune function and cytokine production. J Nutr.

[65] Shankar AH, Prasad AS (1998). Zinc and immune function: the biological basis of altered resistance to infection. Am J Clin Nutr.

[66] Crostarosa F, Aravantinou M, Akpogheneta OJ, Jasny E, Shaw A, Kenney J (2009). A macaque model to study vaginal HSV-2/immunodeficiency virus co-infection and the impact of HSV-2 on microbicide efficacy. PLoS One.

[67] Gianella S, Strain MC, Rought SE, Vargas MV, Little SJ, Richman DD (2012). Associations between virologic and immunologic dynamics in blood and in the male genital tract. J Virol.

[68] Mark KE, Wald A, Magaret AS, Selke S, Olin L, Huang ML (2008). Rapidly cleared episodes of herpes simplex virus reactivation in immunocompetent adults. J Infect Dis.

[69] Tronstein E, Johnston C, Huang ML, Selke S, Magaret A, Warren T (2011). Genital shedding of herpes simplex virus among symptomatic and asymptomatic persons with HSV-2 infection. JAMA.

[70] Corey L, Wald A, Celum CL, Quinn TC (2004). The effects of herpes simplex virus-2 on HIV-1 acquisition and transmission: a review of two overlapping epidemics. J Acquir Immune Defic Syndr.

[71] Buck CB, Thompson CD, Roberts JN, Muller M, Lowy DR, Schiller JT (2006). Carrageenan is a potent inhibitor of papillomavirus infection. PLoS Pathog.

[72] Smith JM, Srinivasan P, Teller RS, Lo Y, Dinh CT, Kiser PF (2015). Tenofovir disoproxil fumarate intravaginal ring protects high-dose depot medroxyprogesterone acetate-treated macaques from multiple SHIV exposures. J Acquir Immune Defic Syndr.

[73] Goode D, Aravantinou M, Jarl S, Truong R, Derby N, Guerra-Perez N (2014). Sex hormones selectively impact the endocervical mucosal microenvironment: implications for HIV transmission. PLoS One.

[74] Lewis RA, Taylor D, Natavio MF, Melamed A, Felix J, Mishell D (2010). Effects of the levonorgestrel-releasing intrauterine system on cervical mucus quality and sperm penetrability. Contraception.

[75] Munch J, Rucker E, Standker L, Adermann K, Goffinet C, Schindler M (2007). Semen-derived amyloid fibrils drastically enhance HIV infection. Cell.

[76] Tan S, Lu L, Li L, Liu J, Oksov Y, Lu H (2013). Polyanionic candidate microbicides accelerate the formation of semen-derived amyloid fibrils to enhance HIV-1 infection. PLoS One.

[77] Barnable P, Calenda G, Ouattara L, Gettie A, Blanchard J, Jean-Pierre N (2014). A MIV-150/zinc acetate gel inhibits SHIV-RT infection in macaque vaginal explants. PLoS One.

[78] Nixon B, Stefanidou M, Mesquita PM, Fakioglu E, Segarra T, Rohan L (2013). Griffithsin protects mice from genital herpes by preventing cell-to-cell spread. J Virol.

[79] D’Cruz OJ, Uckun FM (2006). Dawn of non-nucleoside inhibitor-based anti-HIV microbicides. J Antimicrob Chemother.

